# Reversible normalisation of serum TSH levels in patients with autoimmune atrophic gastritis who received L-T4 in tablet form after switching to an oral liquid formulation: a case series

**DOI:** 10.1186/s12876-016-0439-y

**Published:** 2016-02-24

**Authors:** Poupak Fallahi, Silvia Martina Ferrari, Ilaria Ruffilli, Alessando Antonelli

**Affiliations:** Department of Clinical and Experimental Medicine, University of Pisa, Via Savi 10, 56126 Pisa, Italy

**Keywords:** L-thyroxine, Liquid L-thyroxine, Hypothyroidism, Gastritis

## Abstract

**Background:**

L-thyroxine (L-T4) malabsorption is a potential concern in patients with autoimmune atrophic gastritis.

**Methods:**

We evaluated five patients with autoimmune gastritis, who showed high serum thyrotropin (TSH) levels (in the hypothyroid range) while in therapy with L-T4 in tablet. All patients were switched to receive an oral L-T4 liquid formulation maintaining the same dosage.

**Results:**

In all patients who received L-T4 in tablet form after switching to an oral liquid formulation with the same L-T4 dosage, TSH circulating levels were normalized. In four patients who were switched back again to receive L-T4 in tablets, maintaining the dosage, TSH levels worsened again reaching levels in the hypothyroid range.

**Conclusions:**

The fact that the change from tablets to liquid oral formulation normalised serum TSH levels, and that switching back to tablets caused thyrotropin levels to worsen, leads us to believe that absorption of L-T4 is greater with oral liquid formulations in these patients. These results suggest that the L-T4 oral liquid formulation could circumvent the pH alteration resulting from atrophic gastritis.

## Background

Thyroxine is absorbed through the intestinal mucosa at the level of the jejunum and ileum. Intestinal absorption of thyroxine is influenced by several factors, including the age of the patient, adherence to therapy, dietary habits, absorption kinetics, malabsorption, and interference of other drugs. The role of an acidic environment in thyroxine absorption has been shown [1], even if gastric acid suppression is inconsistently reported to interfere with levothyroxine (L-T4) absorption [2]. The normally acid environment of the stomach becomes altered in patients with gastritis related to *Helicobacter pylori* infection, atrophic gastritis of the body of the stomach, or both [3, 4]. Recently, Centanni et al. showed that the daily requirement of thyroxine was higher (by 22 to 34 %) in patients with *H. pylori*-related gastritis, atrophic gastritis, or both conditions than in the reference group, in patients with multinodular goiter. These results strongly suggest that normal gastric acid secretion is necessary for the effective absorption of oral thyroxine [5, 6]. It is well known that autoimmune thyroiditis and gastritis are frequently associated [7]. Furthermore, it has been shown that L-T4 requirement in patients with autoimmune hypothyroidism and parietal cell antibodies is increased [8].

For the therapy of hypothyroidism, L-T4 is used worldwide in tablet form, but more recently new L-T4 formulations in soft gel capsule or liquid vials have become available [6, 7].

A recent report has shown an increased absorption rate with liquid L-T4 formulation as opposed to tablet form even in patients without malabsorption [9].

Furthermore, it has been shown that the liquid L-T4 is able to solve tablet L-T4 malabsorption in patients who underwent bariatric surgery [10].

However, until now, to the best of our knowledge, no data are present in literature about the effectiveness of a L-T4 liquid formulation in patients with autoimmune gastritis.

Here we report that a new L-T4 liquid formulation is able to normalize serum thyrotropin (TSH) levels in hypothyroid patients in treatment with L-T4 in tablets.

## Methods

We report the results of a retrospective study. Initially we observed, one patient with autoimmune atrophic gastritis with an increase in serum TSH levels while receiving L-T4 in tablet form. This condition was reversibly resolved by switching to the same dose in a liquid oral formulation. We subsequently identified four additional hypothyroid patients with autoimmune atrophic gastritis, with high TSH while receiving L-T4 tablets.

On the whole we evaluated five patients with autoimmune gastritis, who showed high serum TSH levels (in the hypothyroid range) while in therapy with L-T4 in tablet.

All patients were switched to receive an oral L-T4 liquid formulation maintaining the same dosage.

In all patients TSH, free triiodothyronine (FT3), free thyroxine (FT4) were re-evaluated after 1–2 months from the switch from tablet to liquid L-T4.

Subsequently four patients were switched back again from liquid L-T4 to receive L-T4 in tablets, maintaining the dosage, and TSH, FT3, FT4 were re-evaluated after 2 months from the switch.

Since the liquid L-T4 formulation resulted in a better control of TSH levels, all five patients were finally treated with the liquid L-T4, and TSH, FT3, FT4 were evaluated again after 6 months.

Serum FT4 (normal range, 0.8–1.7 ng/dL), FT3 (normal range, 2.3–4.2 pg/mL), and serum TSH (normal range, 0.3–3.5 μIU/mL) were determined in all samples by electrochemiluminescence immunoassay (Roche Corporation, Indianapolis, IN, USA). The concentration of each hormone at baseline, and after the switch, was calculated as a mean of the two samples collected before the L-T4 dose.

The study was approved by the local Ethical Committee. Each patient gave a written consent for participation in the study, and gave consent for publication of these data.

## Results

- Patient 1 was a woman who was receiving stable substitutive therapy for hypothyroidism due to Hashimoto’s thyroiditis. In December 2011 she was euthyroid receiving 110 μg L-T4 daily in tablet form, ingested 30 min before breakfast. One year later, she was about the same body weight, but her thyroid hormone profile revealed subclinical hypothyroidism. She did not receive any other therapy.
- For the presence of persistent and severe dyspsepsia, she underwent a gastroenterological examination that showed the presence of high levels of gastrin (435 pg/mL), and of increased levels of serum parietal cells antibodies, in presence of atrophic gastritis (at gastroscopy), while the search for *H. pylori* or *H. pylori* antibodies was negative.
- We changed the formulation from oral tablets to the liquid formulation, maintaining the same dosage. After 1 month, her TSH levels were in the normal range and she felt better. To confirm the presumed relationship between oral formulation and TSH normalisation, L-T4 was re-administered at the same dosage in tablet form. Once again, serum TSH increased (Table 1). The patient verified medication compliance.

**Table 1:** Thyroid parameters with L-1 T4 tablets, with liquid L-T4 formulation, after switching back to L-T4 tablets, and after 6 months of liquid L-T4 therapy

	Tablet L-T4				Liquid L-T4				Tablet L-T4				Liquid L-T4
	*L-T4*	*TSH*	*FT4*	*FT3*	L-T4	TSH	FT4	FT3	*L-T4*	*TSH*	*FT4*	*FT3*	TSH
	*μg* *tablet*	*μIU/mL*	*ng/dL*	*pg/mL*	μg liquid (months)	μIU/mL	ng/dL	pg/mL	*μg* *tablet* (months)	*μIU/mL*	*ng/dL*	*pg/mL*	μIU/mL
1	110	9.11	1.37	2.64	110	3.29	1.40	2.85	110	6.31	1.15	2.43	2.17
	–	–	–	–	(1)	–	–	–	(2)	–	–	–	–
2	125	7.8	1.12	2.86	125	4.01	1.23	2.91	125	7.23	1.02	2.76	3.25
	–	–	–	–	(2)	–	–	–	(2)	–	–	–	–
3	100	6.7	1.27	2.34	100	2.12	1.32	2.66	100	4.12	1.55	2.56	1.72
	–	–	–	–	(1)	–	–	–	(2)	–	–	–	–
4	150	8.2	0.97	2.51	150	3.15	1.21	3.12	150	5.67	0.94	2.48	2.46
	–	–	–	–	(2)	–	–	–	(2)	–	–	–	–
5	250	60	0.5	–	250	–	–	–	–	–	–	–	–
	–	–	–	–	(1)	3.8	1.19	2.74	–	–	–	–	0.04
	–	–	–	–	200	–	–	–	–	–	–	–	–
	–	–	–	–	(2)	0.05	1.50	2.83	–	–	–	–	–

TSH, normal range 0.3–3.5 μIU/mL; FT4, normal range 0.8–1.7 ng/dL; FT3, normal range 2.3– 4.2 pg/mL

- Patient 2 The patient was a woman with hypothyroidism due to previous radioiodine treatment for Graves’ disease in 2010. After one year from radioiodine, her thyroid hormone profile revealed subclinical hypothyroidism. In March 2011 she started treatment with L-T4 125 μg/daily in tablet form, ingested 30 min before breakfast and in this way, she became euthyroid, and remained euthyroid with the same dosage for about 2 years. In August 2013 she experienced post-prandial dyspepsia. The presence of *H. pylori* antigen in the stool was negative. She was then submitted to esophagus gastroscopy that showed the presence of atrophic gastritis. In December 2013 with the same L-T4 dosage, her thyroid hormone profile revealed subclinical hypothyroidism; she did not receive any other therapy. In January 2014 we changed the formulation from oral tablets to the liquid formulation, maintaining the same dosage. After 2 months, her TSH levels were in the normal range and she felt better. To confirm the presumed relationship between oral formulation and TSH normalisation, L-T4 was re-administered at the same dosage in tablet form. Once again, serum TSH increased (Table 1).

- Patient 3 was a woman who was receiving stable substitutive therapy for hypothyroidism due to Hashimoto’s thyroiditis. In January 2013 she was euthyroid receiving 100 μg L-T4 daily in tablet form, ingested 30 min before breakfast. One year later, she was the same body weight, but her thyroid hormone profile revealed subclinical hypothyroidism. She did not receive any other therapy. She experienced post prandial dyspepsia. The presence of *H. pylori* antigen in the stool and of *H. pylori* antibodies was negative. She was then submitted to esophagus gastroscopy that showed the presence of atrophic gastritis. The patient had also high levels of gastrin (556 pg/mL), and increased levels of serum parietal cells antibodies.
- We changed the formulation from oral tablets to the liquid formulation, maintaining the same dosage. After 1 month, her TSH levels were in the normal range and she felt better. To confirm the presumed relationship between oral formulation and TSH normalisation, L-T4 was re-administered at the same dosage in tablet form. Once again, serum TSH increased (Table 1). The patient verified medication compliance.

- Patient 4 was a woman who was receiving stable substitutive therapy for hypothyroidism due to Hashimoto’s thyroiditis. In December 2012 she was euthyroid receiving 150 μg L-T4 daily in tablet form, ingested 30 min before breakfast. One year later, she was about the same body weight, but her thyroid hormone profile revealed subclinical hypothyroidism. She did not receive any other therapy.
- For the presence of persistent and severe dyspsepsia she underwent a gastroenterological examination and esophagus gastroscopy that showed the presence of increased levels of serum parietal cells antibodies, in the presence of atrophic gastritis (search for *H. pylori* or *H. pylori* antibodies was negative).
- We changed the formulation from oral tablets to the liquid formulation, maintaining the same dosage. After 2 months, her TSH levels were in the normal range and she felt better. To confirm the presumed relationship between oral formulation and TSH normalisation, L-T4 was re-administered at the same dosage in tablet form. Once again, serum TSH increased (Table 1). The patient verified medication compliance.

- Patient 5 was a male, operated in 2005 with total thyroidectomy for papillary thyroid cancer (PTC) (pT3, N0, M0), and subsequently treated with radioiodine. A recombinant human thyroid-stimulating hormone (rhTSh) test in 2006 was negative for recurrence of PTC. From 2008 to February 2011, with a stable dosage of L-T4 in tablets (150 μg/day), TSH was suppressed (< 0.001 μIU/mL), with normal FT4 and FT3 levels. For the presence of persistent dyspepsia in November 2011, he was then submitted to esophagus-gastroscopy that showed the presence of autoimmune atrophic gastritis. The presence of *H. pylori* antigen in the stool was negative. In July 2012 at the endocrinological control, TSH levels were high, in presence of low circulating levels of FT4, and FT3. The L-T4 tablet dosage was gradually increased (from 150 to 250 μg/day L-T4) without a significant correction of hypothyroidism. In November 2012 we changed the formulation from oral tablets to the liquid L-T4, maintaining the same dosage. After 1 month, his TSH levels were in the normal range and he felt better; after 2 months, TSH was suppressed, and it remained stably suppressed in the subsequent controls.
- All patients were finally treated with liquid L-T4, with a good control of TSH values after 6 months of liquid L-T4 therapy (Table 1).

## Discussion

The gold standard replacement therapy for patients with hypothyroidism is levothyroxine. There are requisites to obtain an effective therapy, that are L-T4 products of optimal quality [11], patients’ compliance [12], dissolution of the hormone in the stomach [13], adequate absorption in the intestines [5, 14], and normal metabolism [15]. Several diseases and drugs affect the absorption and metabolism of L-T4 [1]. Gastritis causes L-T4 malabsorption, by altering the gastric juice pH, thereby affecting L-T4 tablet dissolution [8, 16, 17]. Recent studies have shown that vitamin C improves circulating concentrations of TSH, FT4, and total T3 in patients with gastritis and hypothyroidism receiving L-T4 replacement therapy in tablets, probably by increasing solubility of L-T4 in the stomach [18, 19].

In fact, normal gastric acid secretion is necessary for effective absorption of L-T4 [5] by dissolution of tablets, and drug dissolution and solubility may be altered by restrictive procedures that increase gastric pH in the newly created stomach pouch; this may occur in gastric bypass [20]. However a recent report has shown that the liquid L-T4 is able to solve tablet L-T4 malabsorption in patients who underwent bariatric surgery [10].

It has been also shown that the liquid formulation of L-T4 is extremely effective to circumvent the problem of incomplete absorption of the L-T4 caused by proton pump inhibitors [21].

Here, we report normalisation of serum TSH levels in five patients with atrophic gastritis who received L-T4 in tablet form after switching to an oral liquid formulation. In four patients switching back to tablets caused thyrotropin levels to worsen. The fact that the change from tablets to liquid oral formulation normalised serum TSH levels, and that switching back to tablets caused thyrotropin levels to worsen, in absence of any change of L-T4 dosage, leads us to believe that absorption of thyroxine is greater with oral liquid formulations in these patients; the involved mechanisms need to be further studied.

Previous pharmacokinetics studies with liquid L-T4 have shown a clearly faster onset of absorption, compared to tablets (area under the curve from 0 to 2 h greater than 50 %; time to maximum concentration faster by a mean of 30 min), as consequence that liquid formulation does not need gastric dissolution, and enters directly the small bowel where L-T4 is absorbed [22–25].

However the presence of alcohol in the L-T4 liquid formulation could also play a key role in thyroxine absorption. Indeed, oral mucosal is highly vascularised, and drugs that are absorbed through the oral mucosal directly enter the systemic circulation, bypassing the gastrointestinal tract [26]. Additional studies are needed to clarify this interesting point.

## Conclusions

These results suggest that the L-T4 oral liquid formulation could circumvent the pH alteration resulting from atrophic gastritis [6].

It is likely that patients affected by condition that impairs L-T4 absorption (e.g., atrophic gastritis, *H. pylori* infection and proton-pump inhibitors) could benefit from a liquid formulation.

## Consent

Written informed consent was obtained from the patients for publication of these data.

## Availability of supporting data

The datasets supporting the conclusions of this article are included within the article.

## Abbreviations

FT3: free triiodothyronine
FT4: free thyroxine
L-T4: levothyroxine
PTC: papillary thyroid cancer
rhTSH: recombinant human thyroid-stimulating hormone
TSH: thyrotropin
